# Metal–Organic Framework-Based Drug Delivery Systems for Cancer Therapy: A Review

**DOI:** 10.3390/ijms27031548

**Published:** 2026-02-04

**Authors:** Sedigheh Hatami, Khaled Chahrour, Joelle El Fakhouri, Fares Mohammed, Rana Sabouni, Ghaleb A. Husseini

**Affiliations:** 1 Biomedical Engineering Program, College of Engineering, American University of Sharjah, Sharjah P.O. Box 26666, United Arab Emirates; g00092532@aus.edu; 2 Material Science and Engineering Ph. D. Program, College of Arts and Sciences, American University of Sharjah, Sharjah P.O. Box 26666, United Arab Emirates; b00089517@alumni.aus.edu; 3 Department of Chemical and Biological Engineering, College of Engineering, American University of Sharjah, Sharjah P.O. Box 26666, United Arab Emirates; b00092507@alumni.aus.edu (F.M.); rsabouni@aus.edu (R.S.); 4 Department of Biology, Chemistry, and Environmental Science, College of Arts and Sciences, American University of Sharjah, Sharjah P.O. Box 26666, United Arab Emirates; g00093993@alumni.aus.edu

**Keywords:** metal–organic framework, cytotoxicity, synthesis method, drug loading

## Abstract

Cancer remains one of the most significant global health challenges, with conventional treatments limited by side effects and resistance to drugs. The unique properties of metal–organic frameworks (MOFs), which offer high surface areas, tunable structures, and biodegradable properties, make them promising candidates for cancer therapy. This review focuses on MOF-based drug delivery systems for cancer treatment in biomedical applications. This article discusses various synthesis methods, drug-loading strategies, and cytotoxicity considerations. The relationship between the basic chemistry of MOFs and their biomedical applications is elucidated by how each feature directly affects MOF performance in cancer drug delivery. Therefore, this review is a practical and complete guide for researchers working to translate MOFs into effective cancer treatments. Moreover, the role of stimuli-responsive MOFs in cancer therapy is highlighted, along with recent studies demonstrating the effectiveness of MOF-based drug delivery systems. Overall, MOFs offer opportunities for advancing cancer treatment and controlled drug delivery.

## 1. Introduction

The second leading cause of death worldwide, following cardiovascular disease, is cancer [1]. The occurrence of oncogenic mutations in normal cells leads to unrestricted cell division, which is considered the cause of cancer. The tumor develops when rapidly dividing cells accumulate in a specific site, forming a mass that expands, invades adjacent healthy tissues, and metastasizes to other regions. The somatic mutation theory posits that cancer arises from genetic alterations in a single somatic cell, which are transmitted to its daughter cells, leading to clonal expansion of malignant cells [2]. In the United States, an estimated 2,041,910 new cancer cases and 618,120 cancer deaths are projected to occur in 2025 [3]. Due to advancements in treatment and cancer detection, mortality has declined, with approximately four million fewer deaths recorded through 2021 compared to 1991 [4]. Currently, over half of global treatment trials are focusing on cancer therapies where the choice of treatment depends on several factors, such as the type of cancer, cancer site, and cancer severity. Various traditional methods are commercially available, such as surgery, chemotherapy, and radiotherapy. Meanwhile, modern techniques are still in progress, and some are commercially available, such as target therapy, stem cell therapy, immunotherapy, and combination therapies [5,6]. Most conventional cancer treatment strategies still face several challenges, such as surgical tumor removal, chemotherapy, radiotherapy, X-ray therapy, and the difficulty of treatment depending on how advanced the cancer is in its development [5]. Surgery proves to be the most effective traditional method when used at an early stage of cancer development. Also, Radiation therapy has the potential to damage healthy cells, organs, and tissues as a result of radiation exposure [7]. In addition to chemotherapy-related challenges, cancer cells can develop resistance due to an unfavorable tumor microenvironment [5]. The impact of chemotherapy is further limited by factors, including its inability to target tumor cells, its uptake, rapid clearance by the immune system, and subsequent metabolic reactions [5]. Moreover, there are significant obstacles to the use of multiple drug treatments, including multidrug resistance, collateral toxicity to normal cells, and systemic adverse effects, which necessitate alternatives and safer treatment strategies [8,9]. The biggest obstacle to curing cancer is drug resistance and its delivery systems. However, several drugs and treatment approaches have been approved for cancer therapy, aimed at optimizing drug delivery and overcoming drug resistance. Moreover, the conventional cancer treatment is less effective than the recent strategies due to tumor pathology and abnormal architecture of blood vessel tissues [10]. To overcome this obstacle, researchers focused on drug delivery to develop a route for delivering loaded nanocarriers to the tumor site while minimizing side effects. The most commonly used nanocarriers are liposomes, polymeric micelles, gold nanoparticles, dendrimers, solid lipid nanoparticles, magnetic nanoparticles, and metal–organic frameworks [11].

## 2. Metal–Organic Framework (MOF)

Metal–organic frameworks (MOFs) are a novel class of porous hybrid materials classified as organic–inorganic compounds [12]. In biomedical applications, precise control over particle size and morphology is essential, as particles in the size range of 15–200 nm can effectively enter tumors [12]. Recently, reducing MOFs to the nanoscale has led to the development of nanoscale metal–organic frameworks (NMOFs), which retain the structural, chemical, and physical characteristics of bulk MOFs while offering advantages inherent to nanomaterials [12,13].

MOFs are synthesized by coordinating metal ions or clusters with organic ligands to form complex three-dimensional frameworks. Their structure is determined by the length, geometry, and functional groups of the organic linkers, while different metal–ligand combinations yield diverse architectures [13]. Common metal ions such as Zn^2+^, Al^3+^, Ln^3+^, Cu^2+^, and Cd^2+^ form varied coordination geometries, and linkers like carboxylates, amines, nitrates, phosphates, and sulfonates are essential for framework assembly [14]. The reversible bonding between these components enables rearrangement during polymerization, producing highly ordered structures [14].

NMOFs possess high surface area, tunable porosity, crystallinity, and hybrid composition combining carbon-based organic components with metals or metal oxides [13,14]. Their chemical and thermal stability, luminescence, and modifiability make them suitable for applications in gas storage, catalysis, sensing, and drug delivery [15,16,17].

In biomedicine, NMOFs integrate the advantages of organic and inorganic materials, offering biodegradability, high porosity for efficient cargo loading, and chemical versatility for surface modification [18,19]. These properties enable targeted drug delivery and controlled release [13], with promising results in cancer therapy [20] and imaging, including MR and optical modalities [21]. However, challenges remain regarding accumulation and precise release control, which are being addressed in ongoing studies [22]. For biomedical applications, NMOFs must exhibit biodegradability, biocompatibility, high drug-loading efficiency, and controlled drug release, while balancing structural and biological requirements [23].

**Figure 1:**
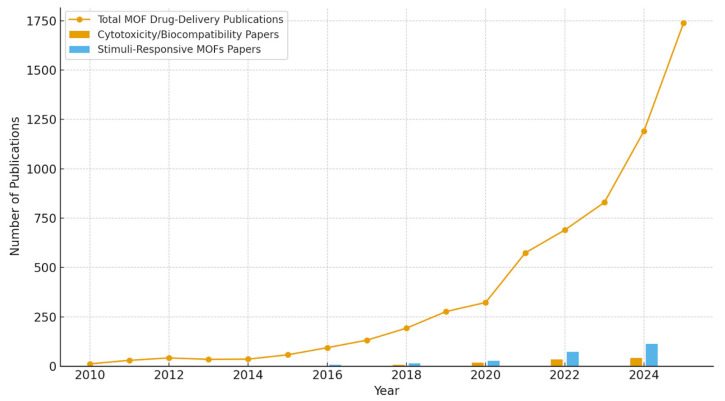
Publication trends and research gaps in MOF drug-delivery studies (2010–2025).

A ScienceDirect analysis was conducted to demonstrate a sharp increase in MOF-based drug-delivery publications, rising from 12 papers in 2010 to more than 1700 in 2025, as shown in Figure 1. This rapid increase reflects the scientific interest in applying MOFs to biomedical applications. The analysis was performed using the ScienceDirect database, where manuscripts were identified through keyword-based searches. The primary keyword “metal–organic frameworks drug delivery” was used to assess overall publication trends. To evaluate specific research areas, additional keywords, including “MOF cancer therapy,” “stimuli-responsive MOFs,” “MOF cytotoxicity,” “MOF biocompatibility,” and “MOF drug loading,” were applied using the advanced search filters, including author-specified keywords. Publications related to stimuli-responsive MOFs and cytotoxicity/biocompatibility were selected by refining search results to include these terms in the title, abstract, or keywords. However, research on these aspects remains limited, with fewer than 50 publications reported in 2024. Additionally, studies on stimuli-responsive MOFs constitute a small fraction of the total number of papers. This imbalance highlights a gap in the literature and underscores the need for a comprehensive review that integrates all the elements addressed in this paper.

## 3. History of Open Framework Coordination

**Figure 2:**
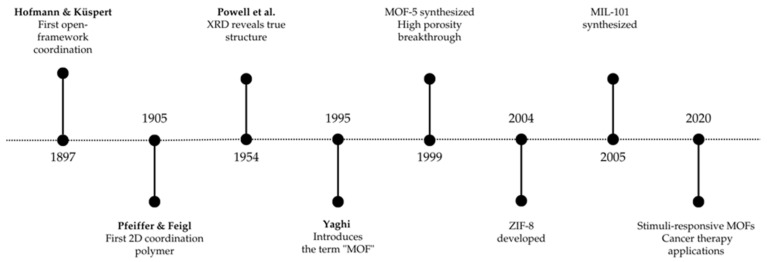
Timeline of major historical milestones in the development of open-framework coordination and MOFs.

The first open-framework coordination was created by Hofmann and Kuspert in 1897. Ni(CN)_2_(NH_3_).C_6_H_6_ crystals were combined in order to create a hybrid network [24]. Later, Pfeiffer and Feigl produced a 2-dimensional polymer with an arrangement involving a single nickel molecule connected to ammonia, two cyanide groups, and a benzene ring, as represented in Figure 2.

In 1954, Powell and his colleagues applied X-ray diffraction (XRD) to characterize Ni(CN)_2_(NH_3_).C_6_H_6_. Contrary to the Hofmann model’s assumption of a 2-dimensional polymer, their findings revealed a structure composed of square flat Ni (II) metal centres enveloped by two cyano groups (CN)_2_ ligands, along with ammonia molecules and a benzene ring contained within these polymer sheets. Figure 2 depicts a partial structure of these network crystals. Subsequently, various research teams endeavoured to modify the Hoffman complex, synthesizing diverse networks using different monomers containing cyano-group ligands and aromatic compounds. According to XRD, the structures of the formed particles were determined to be crystals that encapsulated the aromatic rings [25].

## 4. The First MOFs as Potential Drug Carriers

**Figure 3:**
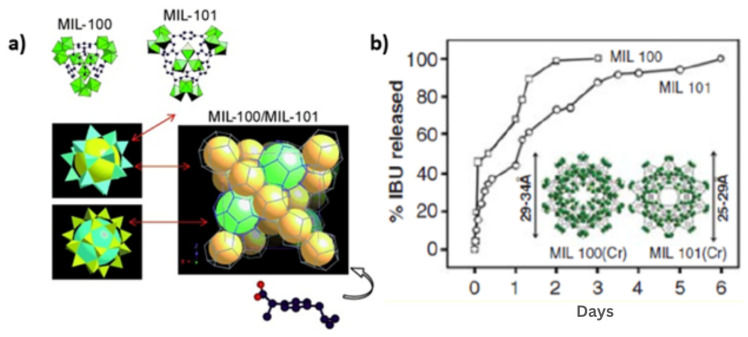
(**a**) represents the MIL-100 & MIL-101 (**b**) represents the Ibuprofen release kinetics from MIL-100 & MIL-101 in SBF at 37 °C [26,27].

In 2006, Horcajada and colleagues investigated the effectiveness of metal–organic frameworks as drug carriers, focusing on MIL-100 and MIL-101, the earliest MOFs suitable for drug delivery. They synthesized these MOFs utilizing carboxylic acid groups and trivalent metals, creating structures with hybrid networks and large pores that are ideal for drug encapsulation [26]. Their study specifically examined the loading and release kinetics of Ibuprofen, an anti-inflammatory drug, within chromium-containing MIL-100 and MIL-101 complexes. MIL-100 was prepared by heating a mixture of metallic chromium, trimesic acid (BTC), hydrofluoric acid, and water. At the same time, MIL-101 was synthesized by heating a mixture of chromium (III) nitrate, hydrofluoric acid (HF), benzene dicarboxylic acid (BDC), and water. Figure 3a illustrates the porous cage-like architectures of MIL-100 and MIL-101, highlighting their large internal cavities that enable high ibuprofen encapsulation. The Ibuprofen loading was achieved through stirring in the presence of the drug, with UV-V spectroscopy indicating high loading efficiencies without compromising the MOF structure. MIL-100 has a surface area of 3100 m^2^/g with a pore size of 25 Å and encapsulates 0.35 g of ibuprofen per gram of MOF. Whereas MIL-101 has a surface area of 5900 m^2^/g with a pore size of 34 Å, encapsulating 1.376 g of ibuprofen per gram of MOF. As shown in Figure 3b, ibuprofen release from MIL-100 and MIL-101 in simulated body fluid (37 °C) occurred over distinct time scales, with complete release achieved within approximately 3 days for MIL-100 and 6 days for MIL-101, reflecting differences in pore size and drug–framework interactions. However, the use of toxic chromium in synthesis limited its usefulness in drug delivery. The study suggested replacing chromium with non-toxic metal ions such as iron, manganese, zinc, copper, or nickel found naturally in the body, thus expanding the potential of MOFs in drug delivery systems (DDS) [26].

## 5. MOF Families

In 1995, Yaghi et al. described the synthesis of a 2D coordination compound using BTC and cobalt, which they named MOF. This marked the formal introduction of the MOF concept. Subsequently, MOFs rapidly developed, giving rise to various MOF families and series. However, there is no standardized naming methodology for MOFs. Names typically reflect the material composition, function, structure, or the laboratory/institution where they were designed, as represented in Table 1 [28]. For instance, the IR in IRMOF-n denotes isoreticularity, and the number n indicates the order of occurrence. Similarly, MIL in MIL-n, refers to the Material of the Institute Lavoisier. Sometimes the numbering system reflects the order of preparation; in other cases, it is arbitrary. For example, MOF-5 is named after the classical zeolite structure ZSM-5 [28]. Below, we discuss the MOF-n family, MIL-n, MTV-MOF, and ZIF-n.

### 5.1. MOF-n Family

In 1999, Yaghi and his team introduced MOF-n, a significant member of the MOF family, particularly for the synthesis of MOF-5, Zn_4_O(BDC)_3_(DMF)_8_(C_6_H_5_Cl). MOF-5 represented a significant advancement in MOF development, showing higher pore volume and surface area than earlier frameworks. It also demonstrated enhanced stability, maintaining its structure after guest molecule removal and withstanding temperatures up to 300 °C [36]. This material was characterized by a hollowness of 55–61% and an aperture of 12.94 Å. Following MOF-5, several MOFs were synthesized, including MOF-1 (1995), MOF-177 (2004), and MOF-74 (2005) [28]. In 2004, Yaghi’s team developed the MOF-177, which displays a 3-dimensional network structure with a narrow pore diameter of 10.8 Å, a 5% hollowness, and an impressive Langmuir surface area of 4500 $\frac{m^{2}}{g}$ far surpassing traditional porous materials. In 2010, they developed MOF-210, boasting a remarkable average porosity of 90% and surface areas of 10,400 $\frac{m^{2}}{g}$ (Langmuir) and 6240 $\frac{m^{2}}{g}$ (BET), and MOF-200 with approximately similar surface areas of 10,400 $\frac{m^{2}}{g}$ (Langmuir) and 4350 $\frac{m^{2}}{g}$ (BET). These advancements in surface areas increase the capacity for drug adsorption and loading, providing promising opportunities for drug delivery systems (DDS) [28].

### 5.2. MIL-Family

The MIL-n family of metal–organic frameworks (MOFs), developed by Férey, is a range of porous materials synthesized at the Institute Lavoisier. Notable members include MIL-100 (Cr, Fe), MIL-101 (Cr, Fe), MIL-53 (Al, Fe, Cr, Ga), and MIL-125 (Ti). These materials offer practical benefits: MIL-88 and MIL-53 exhibit remarkable flexibility akin to sponges with reversible breathing or swelling up to 230% in volume; MIL-100 and MIL-101 provide access to metal active sites and feature hierarchical pore systems [28]. Synthesized using various transition metal elements and dicarboxylic acid ligands, such as succinic acid and glutaric acid, MIL-n MOFs possess the unique ability to transition between micropores and mesopores in response to external stimuli, making them highly versatile materials with potential applications in various fields [28].

### 5.3. MTV-MOF Family

MTV-MOF is a newly proposed metal–organic framework (MOF) derived from MOF-5. These are synthesized by combining two or more diverse organic functional groups into the MOF-5 structure. This enables the use of the unique properties of functional groups such as OC3H5, -NO2, -CH3, -NH2, -OC7H7, -Cl, and -C_4_H_4_ [28]. The positioning, alignment, and proportion of these functional groups along their backbone (phenylene units and metal oxides) are precisely controlled while maintaining the length and connectivity of the linker. Introducing multivariate functional groups is crucial for enhancing the functionality and design of the drug delivery systems (DDS). Also, it facilitates the covalent bonding of drugs, thereby increasing drug loading capacity [28].

### 5.4. ZIF Family

**Figure 4:**
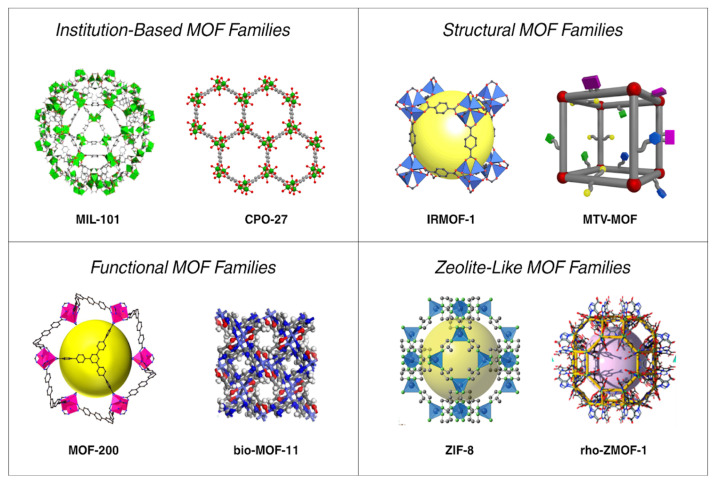
Representation of some MOFs families. (**Top left**): MOFs named after the institution they were discovered in. (**Top right**): MOFs named after their structure and topology. (**Bottom left**): MOFs are named for the functional chemical groups they are made of. (**Bottom right**): MOFs named after Zeolite.

ZIF-n, a notable family of metal–organic frameworks, stands out for its remarkable pH responsiveness, making it ideal for controlled-release applications within a narrow pH range. Moreover, ZIFs exhibit high biocompatibility in vivo and versatile in vitro surface modifiability, making them highly suitable for biomedical applications, particularly for tumor and bone therapy [35]. Among the various ZIFs, ZIF-8, ZIF-90, and ZIF-67 are commonly used in breast cancer treatment. For instance, ZIF-8, known for its superior drug-carrying and pH-sensitive release, has been researched for breast cancer therapy, aiming to minimize side effects and enhance drug compatibility. However, challenges remain in clinical applications due to its low water stability and potential cytotoxicity [35]. ZIF-90, a modified derivative of ZIF-8, exhibits biocompatibility and relatively rapid in vivo degradation, with potential for surface modification to improve breast cancer targeting [35]. However, despite these advantages, ZIF-90 is generally considered less potent and less convenient than ZIF-8 for drug delivery and synthesis applications. Recently, alternative ZIFs have been explored for breast cancer treatment, including ZIF-67, hollow ZIFs, and double-layer ZIFs, each with distinct approaches to breast cancer therapy and promising cell-killing efficacy [35]. Figure 4 provides a schematic overview of the MOF families discussed in this section, classified by their origin, structural topology, functional-group diversity, and zeolite-like characteristics.

## 6. Synthesis Methods

**Figure 5:**
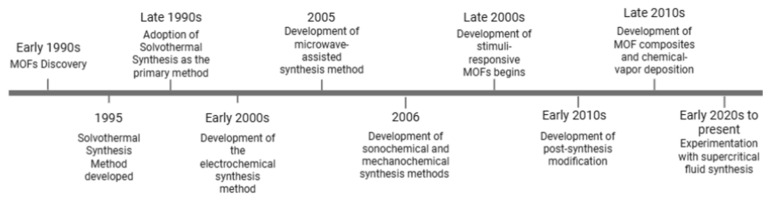
Timeline of development of synthesis methods for metal–organic frameworks.

Several synthesis strategies have been proposed to synthesize MOFs with the required size, surface area, and other properties. Figure 5 provides a chronological overview of the development of major MOF synthesis methods, highlighting how these approaches have evolved to address structural control, efficiency, and scalability. The following sections will discuss various methods for modifying and adjusting MOF forms as alternative methods. A comparative analysis of these synthesis methods, including their key properties, advantages, and disadvantages, is summarized in Table 2.

### 6.1. Solvothermal and Non-Solvothermal Synthesis Methods

**Figure 6:**
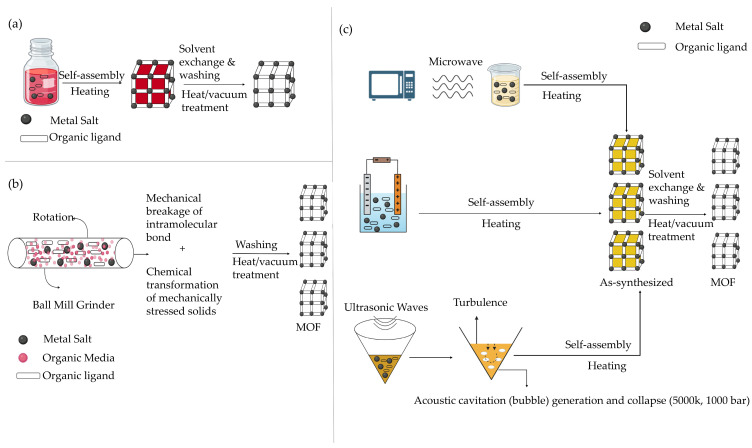
Illustration of MOFs Synthesis Methods (**a**) Solvothermal Method (**b**) Mechanochemical Method (**c**) Microwave, Electrochemical, and Ultrasonic Synthesis Method.

The solvothermal synthesis method relies on heat to produce MOFs; thus, temperature is the key parameter influencing the final structure. The reaction type, solvothermal or non-solvothermal, depends on the nature of the reaction environment. In hydrothermal chemistry, solvothermal synthesis typically occurs in closed reactors at 100–250 °C under elevated autogenous or externally applied pressure [13]. In contrast, non-solvothermal synthesis is conducted in open systems below the solvent’s boiling point and under atmospheric pressure. Generally, the solvothermal method operates under milder thermal conditions than non-solvothermal approaches [13]. Successful MOF synthesis via non-solvothermal routes requires careful selection of metal precursors, organic linkers, solvents, and reaction temperature. Moreover, solvothermal products have more homogeneous structures. A benefit of MOF under solvothermal conditions is that the elements are distributed in a uniform fashion, compared to non-solvothermal processes [13]. An illustration of the solvothermal synthesis route is shown in Figure 6a, and a comparison of the key properties, advantages, and limitations of this method is summarized in Table 2.

### 6.2. Microwave-Assisted Synthesis

Microwave irradiation accelerates the nucleation and growth of MOF crystals by delivering energy through electromagnetic waves. In microwave-assisted synthesis, the movement of ions or polar molecules within the electromagnetic field generates heat, rapidly increasing the reaction temperature and promoting crystal formation [36]. To control crystal growth, it is necessary to control key parameters, such as the solvent nature, energy input, reactant concentration, and reaction time, which determine crystal growth. Microwave-assisted methods have several advantages, including high efficiency, particle size reduction, phase selectivity, and morphology control [36]. Figure 6c represents the microwave-assisted synthesis method.

### 6.3. Electrochemical Synthesis

In electrochemical synthesis, MOFs are produced through the anodic dissolution of a metal anode, generating metal ions that react with dissolved organic linkers and a conducting salt in solution [37,38]. A protic solvent is typically used to prevent metal deposition on the cathode, as hydrogen bound to electronegative atoms (e.g., oxygen) facilitates the formation of hydrogen gas. This continuous process yields a higher solid content compared to conventional batch reactions. The method was first reported in 2005 by BASF researchers for the synthesis of HKUST-1, utilizing bulk copper plates as anodes, H_3_BTC in methanol as the solvent, and copper as the cathode. The synthesis yield and material properties are strongly influenced by parameters such as applied voltage, current density, and temperature [37,38]. It is illustrated in Figure 6c, with its performance characteristics compared with those of other methods in Table 2.

### 6.4. Ultrasonic-Assisted Synthesis

Sonochemical methods use ultrasound to induce homogeneous, accelerated nucleation, thereby reducing crystallization times and yielding smaller particle sizes than conventional solvothermal synthesis. This approach relies on sonochemistry, which involves applying ultrasound frequencies between 20 kHz and 10 MHz, generating acoustic cavitation, as shown in Figure 6c [39]. This process forms, develops, and breaks bubbles in liquids, raising the temperature to (5000–25,000 K) and the pressure to (1000 atm) while producing rapid shock waves. Sonochemical synthesis enhances reaction rates, yields, energy efficiency, and particle quality. Moreover, it offers environmental friendliness, ease of use, applicability at ambient temperature, and a significant reduction in synthesis duration compared to traditional methods [39].

### 6.5. Mechanochemical Synthesis

In this method, mechanical energy is utilized to start the reaction. A chemical change occurs when intramolecular bonds are broken. Mechanical synthesis has the advantage of being solvent-free and possible at room temperature [30]. Furthermore, this synthesis method has a short reaction period and is solvent-free, allowing the use of metal oxide salts instead of metal salts, which serve as precursors, resulting only in water as the byproduct. Metal-oxide salts are the most suitable for use as a precursor since most metal oxides are insoluble [30]. An illustration of this process is shown in Figure 6b.

By comparing the different synthesis methods listed in Table 2, the ultrasonic-assisted (Sonochemical) route is the most suitable for MOF synthesis in biomedical applications. Traditional methods such as Solvothermal synthesis are generally unsuitable for biomedical applications due to the high energy consumption, long reaction times, and the harsh conditions (high temperature and pressure), which compromise the stability of sensitive biomolecules and limit the production of uniform nanoscale particles necessary for effective in vivo drug delivery [13]. However, Mechanochemical and Electrochemical methods offer advantages such as room-temperature operation and solvent-free synthesis; they are limited by poor control over particle size and distribution or high device costs, respectively [31,32,33,34,35,36,37,38]. In contrast, the Sonochemical method is noted as an appropriate process for the synthesis of nanosized MOFs where it operates at low temperature (15–50 °C) with a short reaction time (30–120 min). This combination of nanoscale control, green chemistry principles, and efficiency makes the Sonochemical method the most preferred route for synthesizing biomedical MOFs for cancer treatment. Table 3 summarizes the comparison of the different synthesis methods.

## 7. Drug Loading

**Figure 7:**
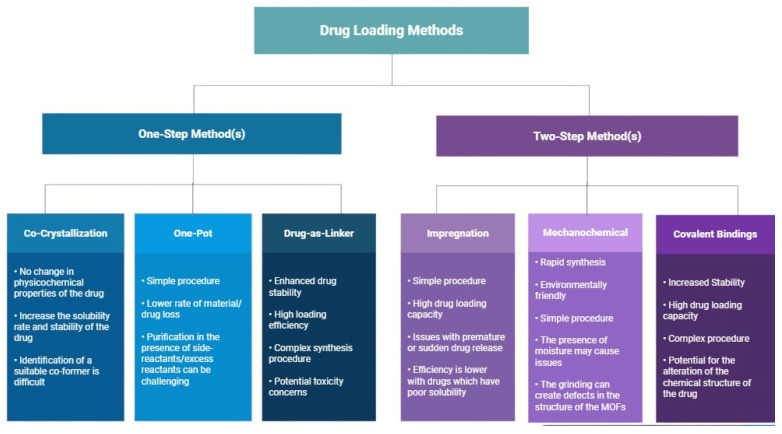
Advantages and disadvantages of drug loading methods. (**Left-side**): Examples, advantages, and disadvantages of some one-step drug loading methods; Co-crystallization, one-pot, and Drug-as-linker methods. (**Right-side**): Examples, advantages, and disadvantages of some two-step drug loading methods; Impregnation, mechanochemical, and covalent binding methods.

Drug loading in metal–organic frameworks (MOFs) leverages their highly ordered structures and large surface areas. Depending on the loading method, drug molecules may adsorb onto the external surface or become encapsulated within the framework’s pores. Two primary loading strategies are commonly used: the one-step and two-step methods [33]. In Figure 7, the main advantages and limitations of the two drug-loading strategies are illustrated for comparison.

Drug loading efficiency in metal–organic frameworks (MOFs) is strongly influenced by their intrinsic properties, including chemical composition, porosity, size, and surface chemistry [32,34]. Larger, highly porous MOFs typically exhibit higher drug-loading capacities due to their greater internal surface area. In addition, precise tuning of pore size and surface functionality can further enhance encapsulation efficiency while minimizing drug loss. Surface modifications, such as adjusting framework charge, organic linkers, or functional groups, are often required to promote specific interactions between the MOF and the drug molecule, thereby improving overall loading performance [32,34].

### 7.1. One-Step

**Figure 8:**
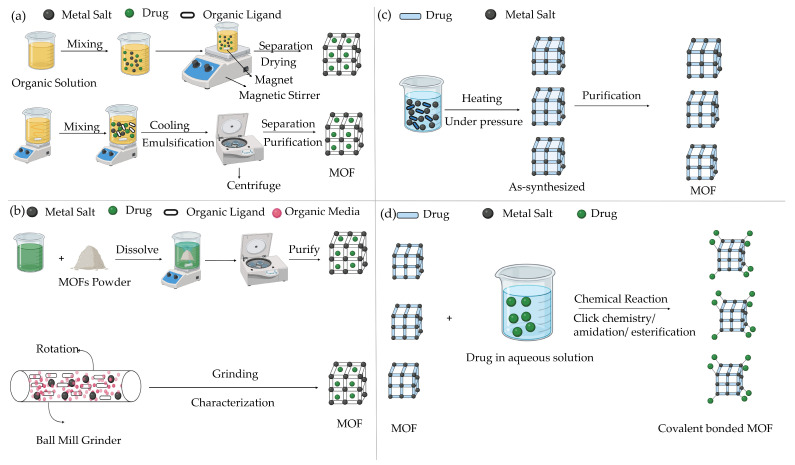
Different MOFs drug loading methods: (**a**) co-crystallization & one-pot method, (**b**) impregnation & mechanochemical methods, (**c**) drug-as-linkers method, (**d**) covalent bonding method.

One-step drug loading strategies involve incorporating therapeutic agents directly during MOF formation, either by embedding the drug within the framework or by using the drug itself as a structural component [40]. This category includes co-crystallization, one-pot synthesis, and drug-as-linker approaches, as illustrated in Figure 8a,c. Compared to post-synthetic loading, one-step methods reduce processing steps and often result in higher loading efficiencies and improved structural integration of the drug [32].

Among these strategies, co-crystallization and one-pot synthesis are the most widely applied. Co-crystallization enables the gentle incorporation of drugs during MOF assembly, preserving their physicochemical properties and enhancing loading capacity. This approach has been successfully demonstrated for a range of pharmaceuticals, including ibuprofen, lansoprazole, leflunomide, and methotrexate, particularly within γ-cyclodextrin-based MOFs [32]. Similarly, the one-pot method allows simultaneous MOF synthesis and drug encapsulation, making it especially suitable for drugs that cannot readily diffuse into pre-formed pores. For example, ZIF-8 MOFs synthesized via a one-pot reaction between Zn(NO_3_)_2_ and 2-methylimidazole in aqueous media exhibited high porosity that enabled efficient loading of 5-fluorouracil (5-FU). Subsequent surface modification through complexation with SDG produced 5-FU@ZIF-8–SDG MOFs, enhancing drug loading and enabling targeted delivery [40]. More broadly, one-pot strategies have been successfully applied to small-molecule drugs such as doxorubicin and 5-fluorouracil, as well as biomacromolecules, where encapsulation within the MOF matrix improves stability and protects sensitive agents, including enzymes, from degradation [33].

In contrast, the drug-as-linker strategy incorporates therapeutic molecules or prodrugs directly into the MOF backbone through coordination with metal nodes. This approach enables exceptionally high drug loading and provides precise control over release behavior. Phosphonate-based drugs coordinated with biocompatible metal ions such as Ca(II) and Mg(II) have been used to construct MOFs that function simultaneously as structural frameworks and drug delivery systems, exhibiting tunable and sustained drug release profiles [33]. Similar strategies have been discussed in the context of BioMOFs, in which biologically active molecules serve not only as therapeutic agents but also as integral components of the MOF structure, allowing the framework itself to function as a drug reservoir [41].

### 7.2. Two-Step

The two-step strategy involves post-synthetic incorporation of drugs into pre-formed MOFs and includes techniques such as impregnation, mechanochemical loading, and covalent binding, as illustrated in Figure 8b,d. These methods are particularly useful when drugs cannot be incorporated during MOF synthesis.

Initially, impregnation involves soaking MOFs in a drug solution so drug molecules can enter through the pores [34]. Various interactions exist between drugs and MOFs, such as Van Der Waals bonding, π-π bonding, and hydrogen bonding, playing a crucial role in the process of binding. Key factors that affect the success of drug incorporation include pore size, window dimensions, chemical composition, and MOF flexibility [34]. MOFs have been used to load caffeine via impregnation, and researchers have embedded drugs such as IBU into porous Cu-MOFs by immersing them in drug solutions [34]. Additionally, Supercritical carbon dioxide has been used to load poorly soluble drugs, such as honokiol, into high-efficiency CD-MOFs. This strategy has also successfully encapsulated phosphate drugs, such as gemcitabine monophosphate, into MIL-100(Fe) with high loading capacity and encapsulation efficiency [34].

Secondly, the mechanochemical method is environmentally friendly and cost-effective for loading drugs. It involves mechanically blending drug powder and MOFs in the solid state without using any solvents [42]. Drugs like 5-FU, caffeine, p-aminobenzoic acid, and benzocaine have been encapsulated into MOFs using this grinding method, achieving high drug loading levels and sustained release [42].

Thirdly, in traditional drug loading methods, various drugs are incorporated into MOFs; however, the weak interactions between the drugs and the framework often result in slow drug release. To address this obstacle, a solution involving covalent bonding and immobilization is needed. Typically, the surface of MOFs has active groups like carboxyl, amino, and hydroxyl, which can form covalent bonds with reactive groups on drugs [43]. For example, Morris et al. demonstrated a DNA-MOF conjugate formed through a click reaction between azide-functionalized UiO-66, dibenzyl cyclooctene-functionalized UiO-66, and dibenzyl cyclooctene-functionalized DNA. This conjugate showed improved stability and cellular transfection capabilities compared to non-functionalized UiO-66 [43]. Similarly, enzymes can be successfully immobilized onto MOFs through covalent binding. Cao et al. reported efficient immobilization of soybean epoxide hydrolase onto the surface of UiO-66-NH2 MOF using a cross-linking approach. These conjugates showed high loading capacity, strong enzymatic-substrate binding, and enhanced catalytic efficiency compared to free enzymes [43].

## 8. Cytotoxicity of MOFs

The toxicity of MOFs introduced into the body must be carefully evaluated and monitored. Several factors influence MOF-induced cytotoxicity, which will be discussed below along with potential mitigation strategies.

### 8.1. Synthesis Method

The synthesis of metal–organic frameworks (MOFs) is significant because it affects their potential toxicity. Traditional methods of making MOFs often involve strong solvents, high temperatures, and hazardous chemicals. This can potentially produce impurities and other substances that might make MOFs harmful to cells. Even after cleaning, some solvents and impurities might still be left behind, making the MOFs even more potentially unsafe [44]. To address this issue, more environmentally friendly methods of producing MOFs have been developed. Those methods may be referred to as green methods, and they focus on utilizing safer solvents, less harsh conditions, and non-toxic ingredients, which help to reduce impurities and other harmful substances [44]. For example, microwaves yield purer, better-formed MOFs at lower temperatures and in less time than traditional methods. Similarly, methods like using sound waves (sonochemical) or grinding (mechanochemical) have been proposed as effective ways to make MOFs, and they have shown promising results in decreasing the risk of toxicity [44].

### 8.2. Pore Size and Core–Shell

The pore size of MOFs can affect their cytotoxicity. If the pores are too small, nutrients and oxygen cannot pass through effectively, causing stress and cell damage. On the other hand, if the holes are too large, immune cells can pass through, causing inflammation that can damage cells [44]. This highlights the critical importance of precisely tuning MOF pore size to achieve an effective drug-delivery system (DDS). Recent studies have demonstrated that MOF cytotoxicity is closely linked to structural features such as pore accessibility and particle size. For example, smaller Fe-based MOFs with more accessible internal surface areas exhibited higher cytotoxicity and oxidative stress in A549 cells than their larger counterparts, underscoring how pore architecture can directly influence responses [45]. Moreover, designing non-toxic MOFs enables the development of core–shell structures in which the core contains the active component and the outer shell is composed of a biocompatible material, thereby reducing overall cytotoxicity [44].

### 8.3. Metal Ion and Organic Part

Furthermore, the types of metal ions and organic molecules used in MOF synthesis can affect their interactions with cells. Some metals utilized, such as cadmium and lead, are known to be toxic to the cells; therefore, choosing a non-toxic metal is a crucial step in MOF synthesis [44]. Similarly, selecting a safe organic linker is important, as some organic linkers can cause cell damage, including cellular stress and inflammatory responses. Recent studies have shown that MOFs are constructed from biologically essential or less toxic metals (such as iron, zinc, or zirconium) and combined with biocompatible organic linkers, generally exhibit lower cytotoxicity compared to MOFs containing heavy or redox-active metals [38]. Moreover, mixed-metal MOFs, in which different metal ions are linked by organic ligands, enable the tuning of pore size and surface properties to enhance biocompatibility [44]. Additionally, the type and microenvironment of the target cells influence the cytotoxic response of MOFs [44]. Hence, it’s crucial to test MOFs across different cell types at varying concentrations to assess their toxicity for medical use.

### 8.4. Natural Material in MOF

Using natural materials in the synthesis of metal–organic frameworks (MOFs) offers advantages over traditional methods. Natural materials are not only cost-effective but also more suitable for medical applications like drug delivery, as they are both biocompatible and non-toxic [44]. However, the use of natural materials presents challenges, as their complex structures and multiple functional groups make it difficult to control the shape and properties of the resulting MOFs [32]. For example, modifying glutathione enables its interaction with zinc ions, leading to the formation of a desired crystal structure, while modification of glucose allows selective interaction with copper ions to achieve a specific framework architecture [44]. Additionally, studies have shown that MOFs constructed from bio-derived ligands or naturally occurring building blocks show reduced cytotoxicity because these materials degrade into biologically tolerable components and minimize harmful metal ion release [46]. Recent advances in sustainable MOF synthesis have explored the use of waste-derived organic ligands (e.g., from PET plastics) and inorganic waste streams as precursor sources, illustrating that alternative, greener building blocks can replace traditional, toxic synthesis reagents and potentially reduce harmful byproducts during production [46].

**Figure 9:**
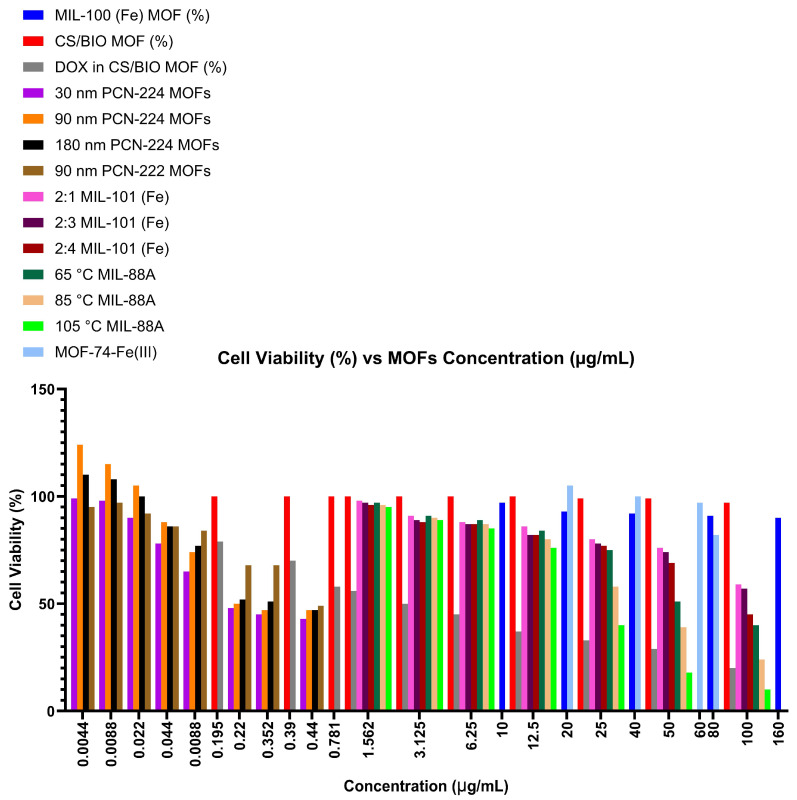
Illustrates the cell viability in % as a function of the MOFs concentration in µg/mL. The data collated from five studies showcases the cytotoxicity of 14 different MOFs used in drug delivery applications. The data is divided across 23 intervals, with the lowest being 0.0044 µg/mL and the highest being 160 µg/mL [37,38,43,44,45,46,47,48]. It is important to note that none of the MOFs were tested at every interval given.

These observations are supported by experimental cytotoxicity data reported in the literature. Figure 9 illustrates the cell viability (%) as a function of MOF concentration (µg/mL) for various MOF systems, demonstrating that cell viability remains largely unaffected for several naturally derived or biocompatible MOFs. For instance, cell viability showed no change across a wide concentration range for CS/Bio-MOFs [37]. Similarly, MIL-100(Fe) and MOF-74-Fe(III) maintained high cell viability values of 89.98% and 82% at concentrations of 160 µg/mL, respectively [44,45]. In contrast, some MOFs exhibited high cytotoxicity at much lower concentrations. For example, 30 nm PCN-224 showed a cell viability of only 40% at a concentration of 0.44 µg/mL, while MIL-88A at 105 °C and 85 °C displayed cell viability values of 10% and 28%, respectively, at their highest tested concentrations [43,46].

### 8.5. Mechanisms of Toxicity and Immune Response

The use of MOFs in cancer therapy requires an understanding of their potential toxic mechanisms and the subsequent immune responses. Although MOFs exhibit a tunable composition and a unique biodegradability, offering a significant advantage over traditional inorganic nanomaterials, their components, the metal nodes and the organic linkers, can trigger a specific cellular pathway that leads to toxicity or immunogenic cell death (ICD) [38]. These pathways are often interrelated and include the generation of reactive oxygen species (ROS), lysosomal dysfunction, and inflammasome activation.

The production of ROS is a primary mechanism of MOF-induced cytotoxicity, leading to oxidative stress, mitochondrial damage, and apoptosis in cancer cells [38]. It is mainly attributed to the nature of the metal-ion clusters in the MOF structure, whereby, upon cellular internalization and degradation, the release of transition-metal ions can catalyze the formation of highly reactive species, such as hydroxyl radicals, via Fenton or Fenton-like reactions.

This mechanism is clearly observed in copper-based MOFs, such as HKUST-1, which is composed of copper (II) paddlewheel units and 1, 3, 5-benzenetricarboxylate linkers. Studies have shown that HKUST-1 triggers mitochondrial membrane depolarization and a high level of ROS in human embryonic kidney cells, directly linking the copper metal node to the oxidative damage pathway [49]. Also, the cobalt-based MOF, such as ZIF-67, which contains cobalt (II) ions and 2-methylimidazole linkers, has triggered apoptosis in microglial cells, a process confirmed to be caused by ROS generation [50]. The toxicity in both cases is linked to the transition metal center’s ability to participate in redox cycling.

Besides the direct induction of oxidative stress, MOFs can lead to toxicity and potent anti-tumor immune responses by disrupting intracellular organelles, specifically the lysosome, which in turn activates the NLRP3 inflammasome. The NLRP3 inflammasome is a multi-protein complex that, when activated, triggers inflammatory responses and a highly immunogenic form of programmed cell death known as pyroptosis [51].

The process begins with the endocytosis of MOF nanoparticles, followed by their accumulation in the acidic lysosomal environment. For MOFs with acid-labile components, such as ZIF-8, the acidic environment causes rapid framework degradation and the subsequent release of the metal component, Zn^2+^ ions. The rapid and strong release of metal ions into the lysosome and cytosol results in ion overload and lysosomal membrane permeabilization (LMP), which leads to lysosomal dysfunction [51]. The released Zn^2+^ ions act as danger signals, triggering the assembly and activation of the NLRP3 inflammasome. This activation cascade culminates in the cleavage of pro-caspase-1 to active caspase-1, which cleaves Gasdermin D (GSDMD) to form pores in the cell membrane, leading to pyroptotic cell death and the release of damage-associated molecular patterns (DAMPs) [51]. This release of DAMPs stimulates a robust anti-tumor immune response, effectively linking the MOF’s toxicity mechanism to its therapeutic immunogenic potential.

## 9. Stimuli-Responsive MOFs

The properties of MOFs make them ideal candidates for drug delivery applications. These properties include their large storage capacity, compatibility with biological systems, and ease of modification [52]. Environmental changes or signals can alter the chemical composition or physical structure of carrier MOFs, facilitating controlled drug release. Studies have shown that MOFs that respond to external stimuli perform better than those that respond to internal cues [52]. For successful drug delivery, MOFs must efficiently load and entrap drugs, be biocompatible, and resist premature drug leakage. Controlled release of small molecules from excipients can be achieved by manipulating environmental conditions [52]. There has been an increase in the use of MOF-based drug delivery systems that are stimuli-responsive in recent years. Stimuli can be internal (pH and redox reactions within the target microenvironment) or external (temperature, light, and magnetic fields) [52]. Single-stimulus-responsive MOFs respond to pH, magnetic fields, ionic interactions, temperature, pressure, light, and humidity. Due to their desirable properties, such as biocompatibility, biodegradability, crystallinity, and porosity, these responsive MOFs are widely used in nanomedicine [52].

**Figure 10:**
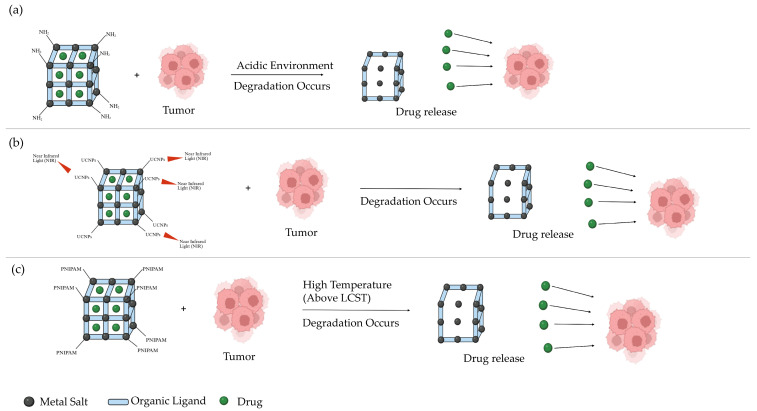
Illustration of MOFs drug release mechanisms (**a**) pH-responsive MOFs: Breakdown of the MOFs and release of the drug in acidic environments (**b**) light-responsive MOFs: Breakdown of the MOFs and release of the drug in the presence of near-infrared light (**c**) thermo-responsive MOFs: Breakdown of the MOFs and release of the drug once exposed to high temperatures.

Recent studies have further demonstrated that stimuli-responsive MOFs significantly increase the efficiency of drug delivery and the therapeutic outcomes in cancer treatment by allowing on-demand and site-specific drug release [46,49]. By responding to tumor-associated internal conditions such as increases in acidity and redox imbalances, these systems reduce off-target drug release and improve drug accumulation at the site of the tumors [46,49]. Stimuli-responsive MOFs have also been shown to increase intracellular drug uptake, enhance cytotoxic effects against cancer cells, and minimize systemic toxicity, making them highly attractive platforms for cancer therapy [53]. Such adaptive behavior allows MOFs to overcome biological barriers and achieve controlled release profiles that are difficult to attain using conventional drug-delivery carriers [54]. Figure 10 illustrates the stimuli-responsive drug-release mechanisms discussed in this section, including pH-, light-, and temperature-responsive MOFs.

### 9.1. pH-Responsive MOF

MOFs with pH responsiveness are an exciting advancement in materials science and nanotechnology. These MOFs have been extensively researched for their ability to react to the acidic environment found in tumor cells [32]. This ability stems from the sensitivity of the coordination bonds in MOFs to external pH. Tumor cells produce large amounts of lactate, lowering the pH of the microenvironment to 6.4–6.9 due to oxygen-independent glycolysis. Figure 10a shows how pH-responsive MOFs are designed to remain stable in physiological conditions, while undergoing structural changes in acidic environments, enabling controlled drug release, thus making them a promising option for cancer therapy in nanomedicine [32].

Recently, pH-responsive MOFs have attracted significant attention for their potential applications in cancer therapy. These systems undergo structural changes under acidic conditions, enabling efficient drug release. Such behavior makes them promising candidates for the oral delivery of anti-inflammatory and chemotherapeutic agents [32]. However, drug release dynamics must be carefully considered during the transition from acidic to intestinal environments [32]. A common design strategy involves using organic ligands with ionizable groups that become protonated in acidic environments while remaining stable at physiological pH (7.4). pH-responsiveness in MOFs is primarily governed by host–guest interactions such as electrostatic forces, hydrogen bonding, and π–π stacking. Among the most studied systems is zeolitic imidazolate framework-8 (ZIF-8), where acidic conditions protonate the organic ligands, leading to cleavage of coordination bonds and subsequent drug release [32]. Other acid-sensitive MOFs (e.g., ZIF-n, MIL-n, UiO-n, DUT-n), acid-labile linkages (amide, oxime, orthoester, imine, polyacetal, polyketide, sulfonate), and pH-sensitive polymers (carboxymethylcellulose, chitosan, gelatin) also degrade in low-pH environments, triggering the release of their payloads [32]. Studies using 5-fluorouracil (5-FU) have demonstrated the high loading capacity and pH-dependent release behavior of ZIF-8, highlighting its promise as a pH-sensitive drug delivery system [32]. In the past year, researchers at the University of Western Ontario in Canada synthesized two pH-responsive Zn-MOF-74 types using zinc acetate and zinc nitrate and coated them with biodegradable polymers, with both exhibiting remarkable drug-release profiles [55]. The MOFs released 40.8% of the drug at pH 5.5, the typical pH of tumor microenvironments [55].

Several studies have demonstrated the effectiveness of pH-responsive MOFs through quantitative in vitro and in vivo evaluations. For instance, Duan et al. developed a pH-responsive MOF-based nanoparticle system for the co-delivery of immunostimulatory CpG oligonucleotides and tumor-associated antigens, achieving approximately 60% antigen release at pH 5.0 [56]. When evaluated in vivo, this system showed increased antitumor activity against B16-OVA melanoma models, highlighting its therapeutic potential in cancer immunotherapy [57]. In a related approach, Pandey et al. designed a hyaluronic acid-coated ZIF-8 MOF system encapsulating 5-fluorouracil and titanocene, which demonstrated pronounced pH sensitivity and improved anticancer efficacy [58]. Drug release reached 92.59 ± 3.5% at pH 5.5 compared to only 18.30 ± 2.7% at physiological pH, while significant reductions in U87MG glioblastoma cell viability were observed after 24 and 48 h of treatment [57]. These studies provide strong experimental evidence that pH-responsive MOFs can achieve selective drug release within acidic tumor environments while maintaining stability under normal physiological conditions.

### 9.2. Light-Responsive MOF

Light is one of the most promising external stimuli for drug delivery systems (DDS) due to its ease of temporal and spatial control. Near-infrared (NIR) light offers better tissue penetration and greater safety than UV light, which has limited tissue penetration [59]. Systems that respond to NIR can be used for targeted drug delivery in clinical settings. When exposed to NIR light, drugs are released via photothermal effects, which causes cell death and enhances drug action, as depicted in Figure 10b. Some mechanisms involve two-photon conversion and upconverting nanoparticles (UCNPs), which can also be used in photodynamic therapy [59]. To combine drug release with photothermal and photodynamic therapy, multimodal carriers have been developed. In photodynamic therapy (PDT), photothermal therapy (PTT), and fluorescence imaging, MOFs have been developed as new building blocks for light-responsive drug delivery management [59]. These include ZIF-8-TNT particles, UiO-66-NH_2_/Ag_3_PO_4_ MOF-nanoparticle composites, nanoscale coordination polymers, ZnPc@ZIF-8 nanocomposites, ICG-CpG@MOF, and DBC-UiO.

In light-responsive MOFs, specific components serve as the primary photothermal agents that convert light into heat. Metallic nanoparticles, particularly gold nanoparticles (Au NPs), are commonly incorporated into MOF structures due to their high photothermal conversion efficiency and relatively low intrinsic toxicity [60]. However, despite these advantages, Au NPs present limitations for clinical translation, including poor biodegradability and a tendency to aggregate in vivo, which may affect long-term safety [61]. In addition to metallic components, organic photoactive linkers within MOFs can also function as light-responsive elements. Recent studies have shown that modifying linker composition can significantly accelerate drug release under light irradiation. For example, MTV-MOFs containing photoactive ligands achieved release rates of up to 0.9 ± 0.1% of the total cargo per minute under green-light excitation, demonstrating rapid and controllable drug release [62]. From a therapeutic perspective, light-responsive MOFs enable both photothermal therapy, in which localized heating induces tumor cell death, and photodynamic therapy, in which light activation generates reactive oxygen species that damage cellular components [63]. While these systems offer precise temporal control, careful optimization is required to balance photothermal performance with framework stability and biological safety.

### 9.3. Temperature-Responsive MOFs

Temperature-responsive DDSs have been widely investigated in cancer treatment. These systems keep drugs stable at normal body temperatures (around 37 °C) and release them at elevated temperatures (above 40 °C) in response to physiological changes or external stimuli [64]. Temperature is among the most convenient and effective triggers for controlled drug release. The temperature difference between cancerous and healthy tissues enables functionalized nanoparticles to release drugs more selectively, as shown in Figure 10c, where temperatures higher than the lower critical solution temperature (LCST) led to degradation of the MOF [29]. Both polymers and MOFs can respond to thermal stimuli; for instance, PNIPAM is a thermo-responsive polymer, while Zr-based MOFs can also release drugs upon temperature changes [54,58]. Oxaliplatin-loaded ZIF-8 MOFs have shown a drug release percentage of 48% by 72 h at an elevated temperature of 40 °C [65]. Furthermore, in combination with a lower pH, specifically 5.5, the cumulative drug release rises to 82% [65].

In temperature-responsive MOF-based systems, thermo-sensitive polymers are typically the key components that sense temperature changes and trigger drug release. Polymers such as PNIPAM and poly(N-vinylcaprolactam) (PNVCL) show LCST behavior in the range of approximately 25–40 °C, undergoing a hydrophilic-to-hydrophobic transition that enables temperature-controlled drug release when integrated with MOFs [66]. Other polymers, including polyethylene glycol (PEG) and poly(acrylic acid) (PAA), provide dual responsiveness to temperature and pH, offering additional control over release behavior [66]. Biologically, tumor tissues can reach elevated local temperatures, especially under hyperthermia or external heating, typically in the range of 40–42 °C. This temperature difference is enough to activate thermo-responsive MOF systems while reducing damage to healthy tissues [67].

## 10. Related Works: Principles, Mechanisms, and Outcomes of MOFs in Cancer Therapy

Metal–Organic Frameworks are a promising class of nanocarriers for cancer therapeutics, primarily due to their structural principle that enables superior drug delivery performance [53,54]. MOFs are well known for their crystalline structure that is formed by self-assembly of metal ions and organic linkers, offering advantages such as ultra-high porosity, large surface area, and chemical tunability [68]. Moreover, the ability to design MOFs for stimuli-responsive drug release is a critical mechanism for enhancing therapeutic outcomes by selectively targeting the tumor microenvironment (TME) [58]. The TME is characterized by conditions such as lower pH, elevated glutathione (GSH) levels, and hypoxia, which can be utilized to trigger on-demand drug release, maximizing efficacy and minimizing systemic toxicity [58]. The following related works demonstrate how various MOF designs express this principle to achieve specific advantages, mechanisms, and therapeutic outcomes.

Yi Li and colleagues investigated the loading of doxorubicin (DOX) onto ZIF-8-on-ZIF-8 nanoplatforms using the MOF-on-MOF approach. This resulted in the creation of DZZ (DOX@ZIF-8-on-ZIF-8) core–shell hierarchical nanostructure. This design illustrates the advantage of structural engineering to boost drug loading capacity. The unique nanoplatforms demonstrated a dual-responsive DOX release, characteristic of a tumor microenvironment (TME). In comparison to conventional monolayer DOX-encapsulated ZIF-8 (DZ), DZZ significantly improved drug loading efficiency by 56-fold and drug encapsulation efficiency by 25-fold [60]. It also improved DOX penetration depth and selectivity, inhibiting breast cancer 4T1 cells in vitro and in vivo. ZIF-on-ZIF nanoplatforms have shown the capability of delivering tumor-targeted drugs and effectively treating breast cancer. This study developed a hierarchical core–shell nanocomposite, DOX@ZIF-on-ZIF, that offers several advantages over conventional monolayer MOF nanoplatforms, including enhanced drug loading capacity, and a dual-trigger mechanism of pH/GSH-controlled drug release under the TME, improved tumor penetration depth, and enhanced therapeutic effects without sacrificing safety compared to free DOX solutions. In addition to contributing to MOF nanostructure design advancement, these engineered nanoplatforms hold promise for both targeted drug delivery and cancer chemotherapy [60]. The key mechanism in this work is the TME-responsive degradation of the ZIF-8 framework, triggered by the acidic pH and high reducing potential GSH found within tumor cells, leading to a controlled and localized drug release.

In a different study, drug delivery systems targeting bones were developed to overcome the limitations of severe adverse reactions to chemotherapy for bone metastatic carcinoma. They created ALN-MOF by combining alendronate (ALN) with doxorubicin (DOX), a widely used anticancer drug, within a metal–organic framework. During in vitro experiments, they assessed the toxicity and uptake of chemicals while studying their pharmacokinetics and biodistribution in vivo [69]. A fast release of DOX was observed at a pH of 5.5 for both MOFDOX and ALN-MOFDOX. When comparing ALN-MOF to MOF, ALN-MOF displayed superior stability, non-cytotoxicity, and enhanced bone affinity [69]. This highlights the advantage of surface functionalization, where the ALN ligand provides a bone-targeting mechanism. Metastases in bone are more likely to accumulate ALN-MOF because it has a prolonged blood retention time. As a result, ALN-MOF can effectively deliver DOX to bone metastases because it’s stable, bone-targeted, and safe [69]. The outcome is a specific delivery system that minimizes off-target toxicity and improves the therapeutic index for bone-related cancers.

Akbar et al. developed versatile bimetallic (FeCo) bi-MIL-88B-FC MOFs modified with folic acid-conjugated chitosan (FC) to deliver the drug 5-Fluorouracil (5-FU) to specific targets. The structure of these nanocarriers was examined using X-ray diffraction and spectroscopy techniques. 5-FU@bi-MIL-88B-FC nanocarriers showed controlled drug release due to the FC coating, releasing a greater amount of drug in acidic environments that mimic cancer cells [70]. In vitro tests demonstrated that the compounds were effective against folate receptor-positive cancer cells with minimal toxicity to healthy cells. The advantage of this system is its dual-targeting mechanism: the FC coating targets folate receptor-positive cells, and the acidic environment triggers the release. Additionally, the nanocarriers exhibited peroxidase-like activity, suggesting potential for comprehensive cancer treatment. This study indicates that 5-FU@bi-MIL-88B-FC is an effective smart drug delivery system for targeted cancer treatment, owing to its sustained release of the drug and its ability to target cancer cells selectively [70]. The study yields a multifunctional nanoplatform that combines chemotherapy with intrinsic therapeutic activity to achieve synergistic effects.

In this study, a multifunctional delivery system called HA-MIL-125@DVMA was developed to target tumor multidrug resistance (MDR) and inhibit both drug efflux and autophagy simultaneously, aiming to prevent treatment failure and tumor recurrence during chemotherapy [71]. This work demonstrates the principle of MOFs as multifunctional platforms for overcoming complex biological barriers. The system employed hollow nanoparticles loaded with doxorubicin-vitamin E succinate (DV) and 3-methyladenine (3-MA) to reverse MDR. DV, which is pH-sensitive, killed tumor cells and inhibited P-glycoprotein (P-gp) mediated drug efflux, while 3-MA inhibited autophagy [71]. The HA-MIL-125@DVMA efficiently released drugs in the tumor microenvironment due to a rapid hydrazone bond-breaking under low pH conditions [71]. In vitro experiments demonstrated that HA-MIL-125@DVMA effectively suppressed drug uptake and inhibited tumor growth without adversely affecting body weight. The combination of acid-sensitive prodrug DV and autophagy inhibitor 3-MA in HA-MIL-125 nanoparticles enhanced antitumor effects and reversed tumor MDR, providing a promising method for treating MDR [71]. The mechanism is a multi-component, pH-responsive co-delivery system, and the outcome is the successful reversal of tumor MDR and enhanced synergistic antitumor effects.

In addition to stimuli-responsive behavior, MOFs have been widely explored for passive and active tumor-targeted drug delivery. Passive targeting primarily relies on the enhanced permeability and retention (EPR) effect, where nanoscale MOFs preferentially accumulate in tumor tissues due to leaky vasculature and poor lymphatic drainage [72]. Building on this concept, recent studies have demonstrated that MOFs can also be engineered to achieve active targeting by incorporating ligands that recognize overexpressed receptors on cancer cells. For example, a heparin (HEP)-functionalized Cu-based MOF was developed for the delivery of doxorubicin (DOX), combining passive accumulation via the EPR effect with active targeting via CD44 receptor-mediated uptake [73]. CD44 receptors are highly expressed on the surface of many cancer cells, allowing HEP-containing MOFs to selectively enter tumor cells and enhance intracellular drug delivery. The use of Cu-based MOFs further contributed to high drug loading capacity, structural stability, and favorable drug release behavior. As a result, this dual-targeting MOF system demonstrated improved cellular uptake and enhanced anticancer efficacy compared to non-targeted systems, highlighting the potential of MOFs to integrate passive and active targeting strategies for more effective cancer therapy [69,70].

MOFs have also been extensively investigated as platforms for drug–drug combination therapy. Due to their high surface area, tunable pore structure, and chemical versatility, MOFs can simultaneously encapsulate different drugs with distinct physicochemical properties and release them in a controlled manner. Several studies have demonstrated that MOF-based co-delivery systems show synergistic anticancer effects, outperforming single-drug formulations [74]. For example, combination-loaded MOFs co-encapsulating doxorubicin and a second chemotherapeutic agent showed significantly increased tumor inhibition compared to either drug alone, showing the benefit of synergistic action [75]. Compared to conventional nanocarriers, MOF-based drug–drug delivery systems provide increased stability, higher loading efficiency, and improved control over release kinetics, making them promising candidates for combination chemotherapy in cancer treatment [74].

MOFs have shown great promise as non-viral carriers for siRNA delivery in cancer therapy by overcoming key challenges associated with naked siRNA, such as rapid degradation, poor cellular uptake, and non-specific distribution. Traditional siRNA molecules are vulnerable to nucleases in biological fluids and often fail to reach target cells in sufficient amounts for effective gene silencing. By encapsulating siRNA within the porous structure of MOFs, these frameworks can protect siRNA from enzymatic degradation. For example, biomimetic MOF nanoparticles loaded with siRNA have been shown to achieve effective tumor suppression in preclinical models by enhancing cellular uptake and ensuring controlled release of the genetic cargo within the tumor microenvironment [75].

## 11. Challenges of MOFs in Drug Delivery

Despite their promising potential in drug delivery, MOFs still face several important challenges that limit their practical use in clinical settings. One of the main concerns includes the environmental impact of MOF production, as many synthesis methods rely on toxic organic solvents such as N,N-dimethylformamide (DMF) and N,N-dimethylacetamide (DMA). These processes also demand high energy input, which not only raises ecological concerns but also slows down the shift toward large-scale manufacturing [44]. Recent studies have also suggested that specific MOF formulations can trigger oxidative stress, mitochondrial damage, and dysfunctional autophagy, which further shows the need for careful toxicological evaluation [76]. Limited structural stability may lead to premature drug release, reducing therapeutic effectiveness [32]. Additionally, scalability remains difficult, since methods such as solvothermal synthesis are often time-consuming, resource-intensive, and challenging to adapt for industrial production [77]. Therefore, these challenges highlight the need for ongoing research into greener synthesis routes, safer material designs, and more robust production strategies before MOF-based drug-delivery systems can be introduced into clinical practice.

### 11.1. Challenges in Clinical Translation: ADME/PK Studies

The preclinical studies show a promising outcome; however, the clinical translation of MOFs is accompanied by complexity and a lack of standardized in vivo absorption, distribution, metabolism, and excretion (ADME) and pharmacokinetics (PK) studies [78]. A major challenge lies in the colloidal stability of MOF nanoparticles within the complex biological environment, where factors like protein corona formation and aggregation can alter their biodistribution and systemic half-life, leading to unpredictable therapeutic outcomes [78]. Furthermore, the lack of a large-scale synthesis protocol and the intricate surface functionalization processes further complicate the generation of reliable and reproducible PK data necessary for regulatory approval [78]. Thus, there is a need for a rigorous and systematic ADME/PK assessment to bridge the gap between preclinical and clinical studies.

### 11.2. MOF Degradation and Clearance

The in vivo fate of MOF represents a critical safety and efficacy consideration that requires systematic assessment, particularly the MOF degradation and subsequent clearance [79]. Although MOFs are often designed for stimuli-responsive degradation within the TME to facilitate drug release, uncontrolled or premature degradation in the systematic circulation can lead to burst release and potential off-target toxicity [78]. The resulting degradation products, which consist of metal ions and organic linkers, must be proven to be non-toxic and efficiently cleared from the body to prevent long-term accumulation and associated adverse effects [80]. Current research is focused on engineering MOFs with predictable and controllable biodegradation profiles by utilizing biocompatible components and incorporating machine-learning-guided computational pipelines to assess the biocompatibility and toxicity of their building blocks [80]. However, understanding the long-term toxicity and the precise mechanisms of clearance is still an ongoing challenge that must be addressed before clinical transition [79].

### 11.3. Nano-Toxicity Assays and Biocompatibility

The evaluation of MOF biocompatibility is crucial for clinical advancement necessitating the adoption of standardized nano-toxicity assays [81]. Recent comprehensive reviews emphasize the need for a systematic toxicity assessment that moves beyond simple in vitro cytotoxicity tests to include complex in vivo models focusing on long-term effects, immunotoxicity, and genotoxicity [44,45]. A significant challenge in this area is the issue of reproducibility, where variations in MOF synthesis, purification, and the choice of control experiments in toxicity assays can lead to conflicting results across different studies [44,46]. Therefore, the field is advocating for standardized protocols for MOF preparation and toxicity testing to ensure reliable data for regulatory bodies [82].

## 12. Future Directions

The trajectory of MOFs in cancer nanomedicine is rapidly evolving toward sophisticated, multifunctional nanoplatforms. One promising direction for MOF-based drug delivery is the development of biodegradable materials. Frameworks constructed from metals naturally found in the body or from metabolizable linkers such as histidine can support safer clearance and minimize long-term tissue accumulation [83]. Advancements in multi-stimuli-responsive MOFs are capable of triggering drug release in response to factors such as pH, redox conditions, light, or enzyme activity, offering greater precision in targeting disease sites and reducing off-target effects [77].

Furthermore, the development of MOF-based hybrid systems is enabling synergistic effects, such as combining the high drug loading capacity of MOFs with the imaging capabilities of other agents [84]. For instance, Quantum Dot (QD)-MOF hybrids are being explored as revolutionary nanoplatforms that enable simultaneous cancer imaging and therapy [84]. Similarly, the development of MOF-gel hybrid systems provides a localized and sustained-delivery approach in which MOFs are embedded within a biocompatible hydrogel matrix to enhance retention at the tumor site [85]. The design of these hybrid systems is increasingly focused on enabling combination therapy.

Moreover, to accelerate the discovery of the optimal structures and predict drug behavior, the integration of artificial intelligence and machine-learning approaches into MOF design is becoming essential [77]. Further research must focus on green and biocompatible synthesis methods to reduce reliance on toxic solvents and high-energy processes, ensuring that MOFs are produced in a more sustainable and clinically acceptable manner. Finally, a comprehensive evaluation of toxicity, biocompatibility, and pharmacokinetics, including absorption, distribution, metabolism, and excretion, is crucial. Addressing these safety mechanisms will be crucial for advancing MOF-based drug delivery systems toward reliable clinical practice.

## 13. Conclusions

Metal–organic frameworks (MOFs) have emerged as a promising alternative to conventional cancer therapies by precisely controlling synthesis methods and drug-loading techniques, and by efficiently functioning as drug-delivery systems that enable targeted and sustained release of therapeutic agents. Moreover, multifunctional and stimulus-responsive MOFs hold great potential for developing personalized cancer treatment strategies. Preclinical studies have demonstrated that MOF-based drug delivery systems can effectively inhibit tumor growth. However, to translate these findings into clinical applications, further research is required to optimize MOF formulations, improve biocompatibility, and ensure safety and efficacy in vivo. With ongoing advancements in MOF technology, the future of cancer therapy appears increasingly promising, offering patients improved treatment outcomes and enhanced quality of life.

## Figures and Tables

**Table 1:** Classification, names, and abbreviations of some MOF families.

Category	Description	Representative Examples	Year of Creation	Refs.
Institution-based	MOFs named after research institutions	MIL-53, MIL-101, CPO-27	2002–2005	[29]
Structural Families	Based on topology or isoreticular design	IRMOF-1(MOF-5), IRMOF-8,MTV-MOF-5	1999–2010	[30,31]
Functional Families	Classified by chemical functionality	MOF-74, RPF-8	2005–2020	[32,33]
Zeolite-like MOFs	Zeolite-inspired frameworks	ZIF-8, ZIF-67, ZMOF-1	2006–2014	[34,35]

**Table 2:** Different synthesis method properties with advantages and disadvantages.

Synthesis Method	Reaction Time	Temperature	Advantages	Disadvantage	Refs.
Solvothermal	24–96 h	50–180 °C	Wide temperature range is beneficial for single crystal High Porosity Reduce Particle size	Involves high expenses to buy High energy consumption Long time Require unique devices (sealed containers, autoclave)	[13]
Microwave-Assisted	5 min–4 h	30–150 °C	Uncomplicated Energy-saving Reduce reaction time Green process Morphology control	Hard to isolate large single crystals Difficult and not easy industrial implementation Expensive device	[36]
Electrochemical	10–60 min	Room Temperature	One-step electrochemical method Particle size control	High device price	[37,38]
Mechanochemical	30–180 min	Room Temperature	Solvent-free synthetic method Only mechanochemical forces are used There is no need for additional temperature and pressure	Single crystals are hard to isolate Limit to specific MOFs It might result in poor crystallinity	[30]
Ultrasonic-Assisted	30–120 min	25–50 °C	Use of high frequency Generating temperature and pressure in a solution by the collapse of bubbles Green process An appropriate process for the creation of nanosized MOFs	Single crystals are hard to isolate	[39]

**Table 3:** Summary of comparison between the different synthesis methods for biomedical applications.

Synthesis Method	Particle Size Control	Solvent Toxicity	Scalability	Suitability for Sensitive Biomolecules	Overall Biomedical Suitability
Solvothermal	2	5	3	2	2
Microwave Assisted	3	3	1	2	3
Electrochemical	4	2	1	5	3
Mechanochemical	2	1	4	5	3
Ultrasonic Assisted (Sonochemical)	5	2	4	4	5

Scale: 1: Very Poor; 2: Poor; 3: Moderate; 4: Good; 5: Excellent.

## Data Availability

No new data were created or analyzed in this study. Data sharing is not applicable to this article.

## Abbreviations

The following abbreviations are used in this manuscript:

ADME	Absorption, Distribution, Metabolism, Excretion
ALN	Alendronate
BDC	Benzene Dicarboxylic Acid
BTC	Trimesic Acid
CMC	Carboxymethylcellulose
DAMPs	Damage-Associated Molecular Patterns
DDS	Drug Delivery System
DOX	Doxorubicin
DV	Doxorubicin–Vitamin E Succinate
DZ	Monolayer DOX encapsulated ZIF-8
DZZ	DOX-loaded ZIF-8 coated with ZIF-8
FC	Folic acid-conjugated chitosan
FU	Fluorouracil
GSDMD	Gasdermin D
GSH	Glutathione
HF	Hydrofluoric Acid
ICD	Immunogenic Cell Death
LCST	Lower Critical Solution Temperature
LMP	Lysosomal Membrane Permeabilization
MDR	Multidrug Resistance
MR	Magnetic Resonance
MIL	Materials of Institut Lavoisier
NMOF	Nanoscale Metal–Organic Framework
NIR	Near-Infrared
PDT	Photodynamic Therapy
PEG	Polyethylene Glycol
PNIPAM	Poly(N-isopropylacrylamide)
PNVCL	Poly(N-vinylcaprolactam)
PTT	Photothermal Therapy
ROS	Reactive Oxygen Species
UCNP	Upconversion Nanoparticles
TME	Tumor Microenvironment
XRD	X-ray Diffraction
5-FU	5-Fluorouracil
3-MA	3-Methyladenine
